# Development and characterization of novel anti‐C5 monoclonal antibodies capable of inhibiting complement in multiple species

**DOI:** 10.1111/imm.13083

**Published:** 2019-06-17

**Authors:** Wioleta M. Zelek, Philip R. Taylor, B. Paul Morgan

**Affiliations:** ^1^ Division of Infection and Immunity School of Medicine Systems Immunity Research Institute Cardiff University Wales UK

**Keywords:** C5, complement, monoclonal antibody, rat, therapeutics

## Abstract

Over the last decade there has been an explosion in complement therapies; one‐third of the drugs in the clinic or in development target C5 protein. Eculizumab, a monoclonal antibody (mAb) that binds C5 and blocks its cleavage by the convertase, is the current reference standard treatment for atypical haemolytic uraemic syndrome (aHUS) and paroxysmal nocturnal haemoglobinuria (PNH) and in clinical trials for many other diseases. Here we describe a panel of novel anti‐C5 mAb, including mAb that, like Eculizumab, are efficient inhibitors of complement but, unlike Eculizumab, inhibit across species, including human, rat, rabbit and guinea pig. Several inhibitory anti‐C5 mAb were identified and characterized for C5 binding and lytic inhibitory capacity in comparison to current therapeutic anti‐C5 mAb; three clones, 4G2, 7D4 and 10B6, were selected and further characterized for ligand specificity and affinity and cross‐species inhibitory activity. The mAb 10B6 was human‐specific whereas mAb 4G2 and 7D4 efficiently inhibited lysis by human, rabbit and rat serum, and weakly inhibited guinea pig complement; 7D4 also weakly inhibited mouse complement *in vitro* The rat C5‐cross‐reactive mAb 4G2, when administered intraperitoneally in a rat model of myasthenia gravis, effectively blocked the disease and protected muscle endplates from destruction. To our knowledge this is the first report of an anti‐C5 function blocking mAb that permits preclinical studies in rats.

**Abbreviations**
Abs: absorbance
AP: alternative pathway
BSA: bovine serum albumin
C5b6: stable complex of C5b and C6
C5: complement component 5
C6: complement component 6
CI: cocktail of protease inhibitors
CP: classical pathway
EAMG: experimental autoimmune myasthenia gravis
ELISA: enzyme‐linked immunosorbent assay
HBS: HEPES‐buffered saline
mAb: monoclonal antibody
MAC: membrane attack complex
NE: neutrophil elastase
PBS: phosphate‐buffered saline
RT: room temperature
ShEA: antibody‐sensitized sheep erythrocytes
WB: Western blot

## Introduction

The complement system is a central component of innate immunity responsible for recognition and killing of bacteria and promotion of phagocytosis through opsonization. The system evolved to protect from pathogens; however, recent reports have highlighted other homeostatic roles of complement, including as a driver of inflammation, modulator of adaptive immunity and metabolism, and architect of neural development.^1^, ^2^, ^3^ Dysregulation of complement leads to pathology, important in a large number of inflammatory and degenerative diseases.^4^ Genome‐wide association studies have provided supporting evidence that complement is involved in many common diseases.^5^, ^6^, ^7^, ^8^ Complement can be the main cause of the disease, for example in paroxysmal nocturnal haemoglobinuria (PNH) and atypical haemolytic uraemic syndrome (aHUS), or contribute to the pathology by mediating tissue injury and inflammation;^4^ in either event, it offers a good therapeutic target.

Over the last decade there has been an explosion in complement drug development. Eculizumab, an anti‐C5 monoclonal antibody (mAb), was a landmark in this work, approved by the US Food and Drug Administration (FDA) in 2007 for use in PNH,^9^, ^10^, ^11^ years later for aHUS^12^ and recently for myasthenia gravis (MG) (news.alexionpharma.com/press‐release/product‐news/fda‐approves‐soliris‐eculizumab‐treatment‐patients‐generalized‐myasthenia). Eculizumab rapidly became the standard treatment for these two ultra‐rare complement‐driven diseases (PNH, aHUS). A host of new agents are in development, targeting the cascade at different stages.^13^, ^14^, ^15^ A recent review listed 34 drugs in the pipeline, many targeting C3 and C5 convertases and the terminal pathway.^14^ C5 occupies a central position in the complement cascade; the C5 convertase cleaves C5 into C5a and C5b, the latter essential for assembly of the membrane attack complex (MAC); inhibition of C5 is therefore an attractive therapeutic target and the focus of several drugs in development.^15^, ^16^ Eculizumab prevents cleavage of C5 by the C5 convertase, blocking generation of the two most inflammatory products of the complement cascade; C5a anaphylatoxin and MAC. The risk of blocking C5 is relatively low; the sole iatrogenic impact of treatment with Eculizumab is an increased risk of Neisserial infections, resolved by vaccination before treatment and prophylactic use of antibiotics.

Despite abundant interest in complement drugs, only three are currently FDA approved; Eculizumab and a recently approved variant engineered for extended half‐life, Ravalizumab (ALXN1210; https://ascopost.com/News/59600), and serum‐derived C1‐Inhibitor marketed as Cinryze or Berinert.^14^ Eculizumab has changed outlook in patients with PNH and aHUS, is now in the clinic for generalized MG, and is in clinical trials for many other diseases. Although effective in these rare diseases, the cost of treatment and high dosage are huge disadvantages for extending use to more common diseases. For example, the annual cost of Eculizumab treatment of a PNH patient is £340 000 in the UK, and the antibody must be administered bi‐weekly by intravenous infusion (900 mg/dose).^4^ There are many agents in development that target C5, for example, SKY59 (RO7112689) an anti‐C5 mAb now in phase II clinical trials. This mAb incorporates a pH‐dependent recycling technology that increases half‐life and decreases dose required for efficient C5 inhibition.^17^ The modified form of Eculizumab, ALXN1210, uses a similar pH switch technology to increase recycling efficiency and drug half‐life.^18^, ^19^ Unlike Eculizumab, RO7112689 also binds C5b6, the intermediate product of MAC formation, and so inhibits reactive lysis.^20^ RO7112689 and Eculizumab were used in this study for comparison with the novel in‐house mAb.

We describe novel anti‐C5 mAb that, like Eculizumab, are efficient inhibitors of complement, but unlike Eculizumab, inhibit efficiently across species, including human, rabbit, rat (clones: 4G2 and 7D4) and weakly guinea pig and mouse (7D4). The species cross‐reactivity of these mAb makes them powerful tools for proof‐of‐concept animal studies. To our knowledge, this is the first report of cross‐species blocking anti‐C5 mAb. One of these mAb was shown to efficiently inhibit complement *in vivo* in rats and to prevent disease in a rat model of MG (passive transfer experimental autoimmune MG; EAMG). Surface plasmon resonance (SPR) analysis of selected mAb demonstrated strong and stable binding to both human and rat C5, making these antibodies very strong candidates for tool therapeutics.

## Materials and methods

All chemicals, except where otherwise stated, were obtained from either Fisher Scientific UK (Loughborough, UK) or Sigma Aldrich (Gillingham, UK) and were of analytical grade. All tissue culture reagents and plastics were from Invitrogen Life Technologies (Paisley, UK). Sheep and guinea pig erythrocytes in Alsever's solution were from TCS Biosciences (Claydon, UK). Eculizumab was kindly donated by Prof. David Kanavagh (Newcastle University, UK), and RO7112689 by Roche Diagnostics (Basel, Switzerland).

Human and animal sera were prepared in house from freshly collected blood. For human, rabbit, rat and guinea pig, blood was clotted at room temperature for 1 hr, and then placed on ice for 2 hr for clot retraction before centrifugation and harvesting of serum. For mouse, blood was placed on ice immediately after harvest and clotted for 2 hr on ice before serum harvest. Sera were stored in aliquots at −80° and not subjected to freeze–thaw cycles.

#### Generation of anti‐C5 mAb

Mouse mAb to C5 were generated by immunization of C6‐deficient mice (bred in house) with C5b6 using standard schedules.^21^ C5b6 was used as immunogen to increase the likelihood of obtaining function‐blocking mAb. C6‐deficient mice were derived from a spontaneous C6‐deficient mouse,^22^ back‐crossed eight generations onto C57BL/6. C5b6 was prepared in house by incubating C5 and C6 with a fluid‐phase convertase comprising cobra venom factor and activated factor B; the complex was then purified by gel filtration. Immunized mice were screened for antibody responses by enzyme‐linked immunosorbent assay (ELISA), mice with the highest titre response were selected and re‐boosted before killing and harvesting of spleens. Plasma cells were harvested, fused with SP2 myeloma and aliquots were placed in 96‐well plates. Positive hybridomas were selected by direct ELISA on immobilized C5b6 and by haemolysis assay for blocking activity as described below. C5b6‐positive complement inhibitory mAb‐secreting clones were sub‐cloned by limiting dilution to monoclonality. Mouse mAb were isotyped using IsoStrips (# 11493027001; Roche). Over multiple fusions, approximately 864 000 hybridoma clones were screened, 139 antibodies were selected from ELISA, 12 were confirmed to be inhibitory, and three of these, 4G2, 7D4 and 10B6, were chosen for full characterization.

#### Haemolytic assays

The inhibitory activity of mAb in human and animal sera was investigated by classical pathway (CP; CH50) haemolysis assay using antibody‐sensitized sheep erythrocytes (ShEA) or alternative pathway (AP; AH50) assays using rabbit erythrocytes (RabE); animal blood was from TCS Bioscience and anti‐ShE antiserum (#ORLC25, Siemens Amboceptor) was from Cruinn Diagnostics (Dublin, UK). ShEA were suspended in HEPES‐buffered saline (HBS) containing Ca^2+^ and Mg^2+^ at 2% (vol : vol), RabE in HBS containing 5 mm EGTA and 3 mm MgCl_2_.^23^ For measurement of CP activity in male mouse serum, ShEA were additionally incubated with mouse anti‐rabbit IgG at 25 μg/ml (#3123; Invitrogen) for 30 min at 37° before washing in HBS. A serial dilution series of each test mAb (100–0 μg/ml; 50 μl/well) was prepared in HBS and aliquoted in duplicate into a 96‐well round‐bottomed plate at 50 μl/well, then serum and 2% ShEA (50 μl/well of each) added. Serum dilutions for each species were selected in preliminary experiments to give near‐complete haemolysis in the CP assay in the absence of test mAb: normal human serum, 2·5%; normal male mouse serum, 25% (using the double‐sensitized cells as described above), normal rat serum, 2·5%; normal guinea pig serum, 2·5%; normal rabbit serum, 25%. Plates were incubated at 37° for 30 min, centrifuged and haemoglobin in the supernatant was measured by absorbance at 405 nm. Percentage lysis was calculated according to: % Lysis = Absorbance (Abs) sample – Abs background)/(Abs max − Abs background) × 100%. graphpad prism was used for data analysis. Hybridoma supernatants were screened for blocking mAb using the same assay but with neat tissue culture supernatant in place of the purified mAb. C5a generation in CP haemolysis assay supernatants was measured using an ELISA kit (# HK349‐02; Hycult Biotech, Uden, the Netherlands).

#### Characterization of novel mAb by ELISA

Direct ELISA and sandwich assay were used to test whether the new mAb bound C5, C6 or C5b6. Sandwich ELISA were used to confirm C5 binders to eliminate issues around denaturation and demonstrate separate epitopes for test mAb to RO7112689 used as capture. Standard curves were generated using in‐house proteins; C5, C5b6 and C6 purified as previously described^20^, ^23^.

In the direct ELISA, Maxisorp (Nunc, Loughborough, UK) 96‐well plates were coated with C5, C5b6 or C6 (0·5 μg/ml in bicarbonate buffer, pH 9·6) at 4° overnight; wells were blocked [1 hr at 37° with 2% bovine serum albumin (BSA) in phosphate‐buffered saline (PBS)], washed in PBS containing 0·05% Tween‐20 (PBS‐T). Dilutions of purified mAb, 1000–0 ng/ml (stock concentrations of all proteins used established using the BCA assay) in 0·2% BSA‐PBS, were added in triplicate to wells coated with each of the three antigens and incubated for 1 hr at 37°. Wells were washed with PBS‐T then incubated (1 hr, 37°) with secondary antibody (donkey anti‐mouse‐horseradish peroxidase (HRP); Jackson ImmunoResearch, Ely, UK) for 1 hr at 37°.

In the sandwich ELISA, Maxisorp plates were coated with RO7112689 or Eculizumab (1 μg/ml in bicarbonate buffer, pH 9·6) at 4° overnight; wells were blocked (1 hr at 37° with 2% BSA‐PBS) and washed in PBS‐T. Standard curves of in‐house purified proteins; C5, C5b6 and C6 diluted in 0·2% BSA‐PBS, were added in triplicate and incubated for 1 hr at 37°. Wells were washed with PBS‐T then incubated (1 hr, 37°) with in‐house mAb anti‐C5 (1 in 1000 dilution in PBS‐T), followed after washing with HRP‐labelled anti‐mouse IgG as above.

After washing, plates were developed using *O*‐phenylenediamine dihydrochloride (OPD, SIGMAFAST™; Sigma‐Aldrich) and absorbance (492 nm) was measured. graphpad prism was used for data analysis. Assays detection limits, working ranges and assay performance were determined as described.^24^

#### Characterization of mAb by Western blot

C5, C5b6 and C6 (in house; 1 μg) were placed in separate wells and resolved on 4–20% sodium dodecyl sulphate–polyacrylamide gel electrophoresis gels (#4561093; Biorad, Hemel Hempstead, UK) under reducing and non‐reducing conditions, then electrophoretically transferred onto 0·45‐μm nitrocellulose membrane (GE Healthcare, Amersham, UK). After transfer, non‐specific sites on the membrane were blocked with 3% BSA in PBS‐T. After washing in PBS‐T, membrane strips were incubated overnight at 4° with individual test mAb (clone 4G2, 7D4, 10B6; each at 1 μg/ml in 3% BSA PBS‐T) or controls RO7112689 (1 μg/ml) or polyclonal (goat) anti‐human C5 (CompTech, Tyler, TX; A220; 2 μg/ml). After washing, bound test mAb were detected by incubation with donkey anti‐mouse IgG‐HRP (715‐035‐150; Jackson ImmunoResearch), RO7112689 with donkey anti‐human IgG‐HRP (709‐036‐149; Jackson ImmunoResearch) and polyclonal anti‐C5 with rabbit anti‐goat IgG HRP conjugate (305‐035‐045; Jackson ImmunoResearch) at 1 : 5000 in PBS‐T. After washing, the blot was developed with enhanced chemiluminescence (GE Healthcare) and visualized by autoradiography.

#### Testing impact of mAb on atypical cleavage of C5 by neutrophil elastase

C5 was mixed with test mAb at 5× molar excess in HBS, then incubated for 15 min at room temperature. Neutrophil elastase (NE, # 16‐14‐051200; Athens Research, Athens, GA) was added at 420 nm final concentration, incubated for 30 min at 37° in a shaking water bath, and the reaction was stopped by addition of 61 nm protease cocktail inhibitors (CI, #P8340; Sigma Aldrich). C5 cleavage was determined by measuring generation of C5a, detected by WB and by ELISA (# HK349‐02; Hycult Biotech). For WB, samples were diluted 1 in 2 in HBS, separated and transferred as above. Blots were probed with anti‐C5a mAb (#HM2079; Hycult Biotech), detected using donkey anti‐mouse IgG‐HRP (Jackson ImmunoResearch, 715‐035‐150). Positive controls included intact C5 (in house), C5a (Comptech), NE and CI diluted in HBS.

#### SPR analysis to determine test mAb binding affinity to human and rat C5

The human C5 mAb binding analyses were carried out on a Biacore S200, whereas rat C5 mAb binding was tested using a T200 instrument (GE Healthcare); in both, a Mouse Antibody Capture kit (# BR‐1008‐38; GE Healthcare) was used to immobilize capture antibody on a CM5 sensor chip (#29‐1496‐03; GE Healthcare) at RU = 500 (for human C5 testing) or 400 (for Rat C5 analysis). Test mAb were flowed to saturate the surface, then C5 proteins (human or rat) in HEPES‐buffered saline (10 mm HEPES, pH 7·4, 150 mm NaCl, 0·005% surfactant P20) flowed over the immobilized mAb. For kinetic analysis, the flow rate was maintained at 30 μl/min, and data were collected at 25°. Data from a reference cell were subtracted to control for bulk refractive index changes. The *R*_max_ was kept low and the flow rate high to eliminate mass transfer. All reagents used were of high purity, polished by size exclusion chromatography immediately before use to ensure removal of any aggregates. Data were evaluated using the software appropriate to the instrument (biacore evaluation Software, GE Healthcare).

#### Passive transfer EAMG in rats

To test *in vivo* effects and therapeutic efficacy of test mAb, Wistar Han IGS rats (100–150 g) were obtained from Charles River Laboratories (Edinburgh, UK) and allowed to acclimatize for 1 week before disease induction by intraperitoneal administration of anti‐Acetylcholine receptor (AChR) mAb35 at 1 mg/kg in PBS as described previously.^25^ mAb35 binds the main immunogenic region of AChR, activating complement and damaging the neuromuscular junction endplates, causing severe muscle weakness. Animals were assessed hourly post‐disease initiation as described previously.^25^, ^26^ mAb35‐injected rats were split into two groups: Group one (*n* = 4) was treated with test mAb at 40 mg/kg dose (selected dose determined in a pilot study) subcutaneously at time zero, with a second subcutaneous dosage of 13 mg/kg at 24 hr. The second group (*n* = 3) received an irrelevant isotype control antibody at the same times, routes and doses. All animals were killed at 40 hr post‐induction, blood was taken for serum assays, soleus muscles were harvested and frozen in OCT mounting medium for sectioning as described previously.^25^, ^26^ Sections were fixed in ice‐cold acetone for 15 min at −80° and then blocked for 30 min in 10% horse serum/2% BSA. After washing in PBS, sections were stained overnight at 4° with primary antibodies, C3/30 anti‐C3b/iC3b mAb (in house) at 10 μg/ml, and rabbit anti‐rat C9/MAC polyclonal IgG (in house) at 50 μg/ml, both in the block buffer. Anti‐C3b/iC3b sections were washed and incubated for 15 min at room temperature with amplifier antibody goat anti‐mouse (VectaFluor DyLight 488, # DK‐2488; Vector Labs, Peterborough, UK). After washing, secondary antibody, horse anti‐goat IgG – Alexa Fluor 488 (DyLight 488, # DK‐2488) for C3b/iC3b or goat anti‐rabbit‐FITC (#45002; Oxford Biomedical Research, Rochester Hills, MI, USA) for anti‐C9/MAC were added as appropriate, together with *α*‐bungarotoxin‐TRITC (BtX) (labels AChR; Boitum, # 00012) at 0·5% and Hoechst stain 1 : 10 000 (# 62249; ThermoFisher), then incubated 40 min at room temperature in the dark. Sections were washed in PBS and mounted in VectorShield Vibrance (#H‐1700‐2; Vector Labs) before analysis using an Apotome fluorescence microscope (Zeiss Apotome Axio Observer microscope, Carl Zeiss Microscopy, Cambridge, UK). Ten fields were captured from comparable regions of muscle in each sample at the same exposure and magnification (×40) and the number of BuTx‐reactive endplates in each section was measured using density slicing in an image analysis system (imagej, University of Wisconsin‐Maddison, Maddison, WI, USA). For co‐localization of complement activation products, sections were additionally imaged on a Zeiss confocal microscope (Zeiss LSM800 confocal laser scanning microscope).

## Results

### Cross‐species complement inhibition by mAb in haemolysis assays

**Figure 1:**
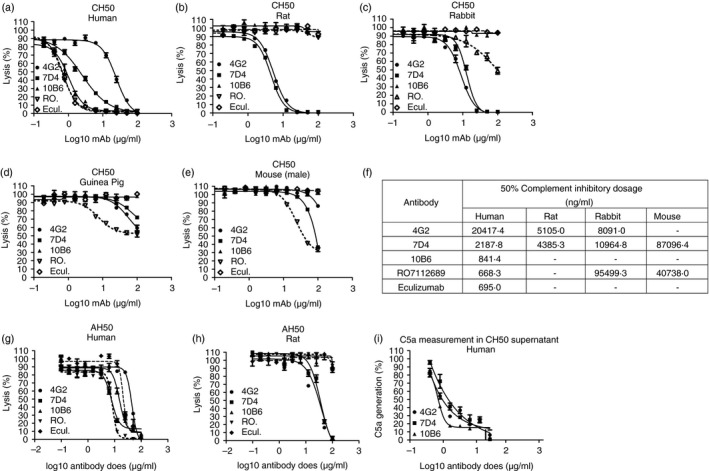
Functional assays to determine whether monoclonal antibodies (mAb) 4G2, 7D4 and 10B6 inhibit complement in different species. (a–e) Classical pathway haemolysis (CH50). Sera tested were human (a), rat (b), rabbit (c), guinea pig (d) and mouse (e). Commercial mAb RO7112689 and Eculizumab were used as comparators. (f) Calculation of 50% inhibitory dose showed that human C5 inhibition by mAb 10B6 was equivalent to the two comparator mAb, RO7112689 and Eculizumab, and that 4G2 and 7D4 strongly inhibited rat C5. (g, h) Alternative pathway (AP) haemolysis (AH_50_) assay using human (g) and rat (h) serum; all tested mAb inhibited AP in human serum, while 4G2 and 7D4 inhibited rat AP. (i) Inhibition of C5a generation by the novel mAb in a classical pathway assay with human serum; all three mAb efficiently inhibited C5a generation in a dose‐dependent manner. All experiments were repeated three times with the same results. The error bars are standard errors of triplicates. The dashed lines correspond to the comparator mAb.

Hybridoma clones were initially selected for further characterization based upon the capacity of clone supernatants to cause inhibition of CP haemolysis of ShEA by human serum; three of the selected monoclonal clones; 4G2 (IgG2b, *K*), 7D4 (IgG2b, *K*), 10B6 (IgG1, *K*), were expanded for further characterization, secreted mAb purified and further tested in haemolysis assays. As expected, each of the selected mAb efficiently inhibited normal human serum‐induced CP haemolysis, with clone 10B6 showing equivalent dose–response performance to the benchmark mAb RO7112689 and Eculizumab (Fig. 1a). When the impact of the mAb on CP haemolysis induced by other species sera was tested, mAb 4G2 and 7D4 efficiently inhibited haemolysis mediated by either rat or rabbit serum (Fig. 1b,c) and weakly inhibited haemolysis by guinea pig and mouse sera (Fig. 1d,e). In contrast, neither the new mAb 10B6 nor Eculizumab inhibited any of these non‐human sera, although RO7112689 inhibited mouse, guinea pig and rabbit but not rat serum‐induced haemolysis. The calculated 50% complement inhibitory doses of all mAb are shown (Fig. 1f); guinea pig serum is not included in this summary because levels of inhibition did not reach 50% for any of the mAb. The novel mAb 10B6, 4G2 and 7D4 also showed efficient inhibition of AP lysis in human serum (Fig. 1g), whereas 4G2 and 7D4 efficiently inhibited rat AP (Fig. 1h). C5a release was inhibited by the novel mAb at the same levels as MAC generation (66 nm) in the human CP assay (Fig. 1i).

### Binding of mAb to C5 and C5b6

**Figure 2:**
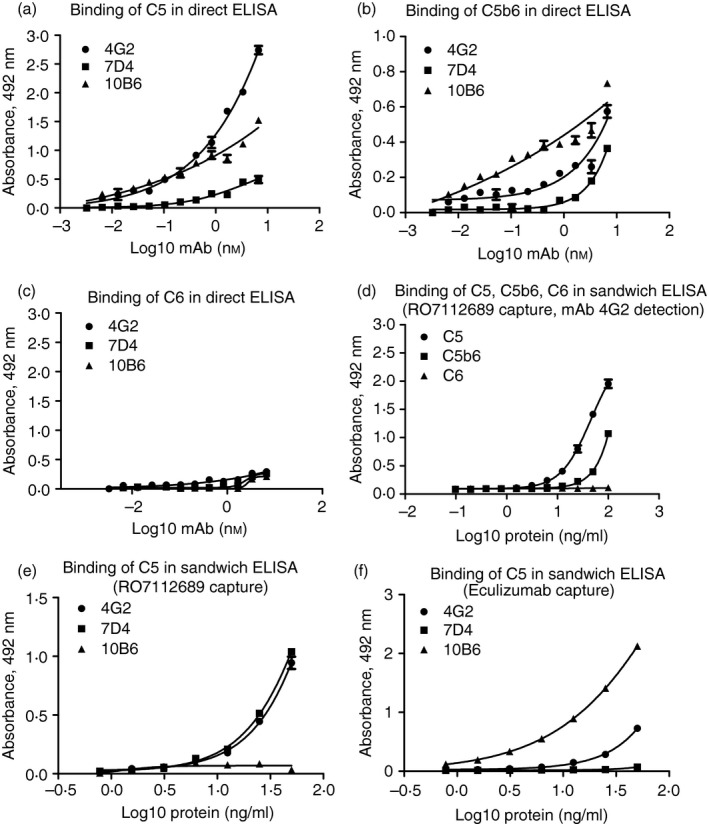
Direct and sandwich ELISA for characterization of new monoclonal antibodies (mAb). In direct ELISA, plates were coated with human C5 (a), C5b6 (b) or C6 (c). All new mAb detected C5 and C5b6 complex, but not C6. In a sandwich ELISA with RO7112689 as the capture and in house 4G2 mAb as detection (d), both C5 and C5b6, but not C6 were detected showing that mAb 4G2 and RO7112689 antibodies recognized different epitopes in C5. Sandwich ELISA comparing RO7112689 (e; anti‐C5*β*) and Eculizumab (f; anti‐C5*α*) as capture mAb showed that both the C5*α*‐specific mAb (7D4 and 4G2) bound strongly to RO7112689‐captured C5 but the C5*β*‐specific 10B6 did not bind (e); the *β*‐chain‐specific mAb 10B6 bound most strongly to Eculizumab‐captured C5 whereas the anti‐C5*α* mAb bound weakly (4G2) or not at all (7D4), suggesting that they competed for similar epitopes (f). All experiments were repeated three times with the same results. The error bars are standard errors of triplicates.

The direct ELISA showed that all the selected new mAb recognized C5 and C5b6 and none recognized C6 (Fig. 2a–c). In a sandwich ELISA with RO7112689 (anti‐C5*β*) as capture and 4G2 mAb as detection, C5 and C5b6, but not C6, were detected; demonstrating that mAb 4G2 and RO7112689 antibodies recognized different epitopes on C5 and C5b in the C5b6 complex (Fig. 2d).

**Figure 3:**
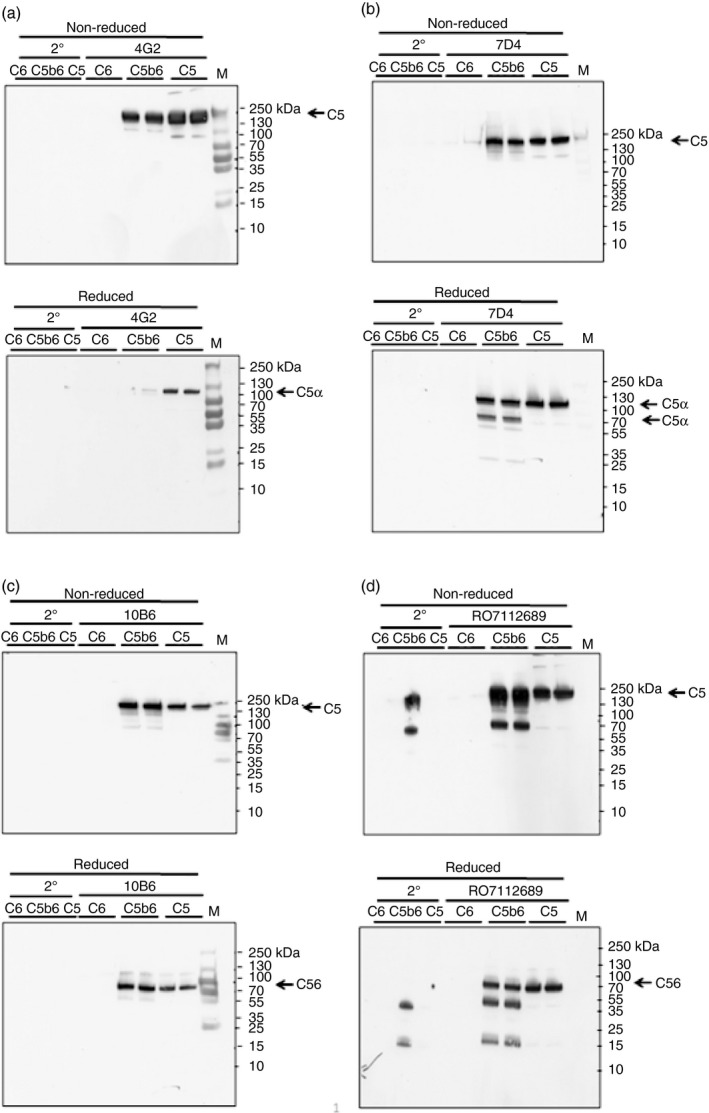
Western blot (WB) using novel monoclonal antibodies (mAb; 4G2, 7D4, 10B6) in comparison with anti‐C5 mAb RO7112689. Proteins; human C5, C5b6 and C6 (1 μg) were resolved on 4–20% PAGE gels under non‐reducing (NR) and reducing (R; 5% *β*‐mercaptoethanol) conditions. Blots were probed with mAb 4G2 (a), 7D4 (b), 10B6 (c), RO7112689 (d). All mAb detected intact C5 and C5b but not C6 in the C5b6 complex. The mAb 4G2 and 7D4 bound the C5*α* chain, while mAb 10B6 and RO7112689 bound the C5*β* chain. All mAb except 4G2 detected C5b6 in reducing conditions. In‐house produced C5b6 probed with RO7112689 showed trace contaminating human antibody bands from the immunoaffinity purification process detected by the anti‐human IgG secondary. Results are representative of multiple analyses.

To confirm which chains of C5 the new mAb bound, we performed Western blotting on purified C5. The blots demonstrated that mAb 4G2 and 7D4 bound the *α*‐chain in C5; of note, although 7D4 recognized both residual *α*‐chain and the *α*′‐chain in C5b6, 4G2 only bound residual intact *α*‐chain (Fig. 3a,b). 10B6 recognized the *β*‐chain of C5 (Fig. 3c); RO712689 also recognized the *β*‐chain (Fig. 3d), whereas Eculizumab is known to bind the macroglobulin 7 domain in the *α*‐chain.^27^ Polyclonal anti‐C5 recognized both C5 *α*‐ and *β*‐chains (not shown).

To test whether the novel anti‐C5 mAb recognized epitopes distinct from the commercial mAb, sandwich ELISA were developed using RO7112689 or Eculizumab as the capture mAb. With RO7112689 as capture, both the C5*α*‐specific mAb (7D4 and 4G2) bound strongly but the C5*β*‐specific 10B6 did not bind (Fig. 2e). With Eculizumab (anti‐C5*α*) capture, the *β*‐chain‐specific mAb 10B6 bound most strongly whereas the anti‐C5*α* mAb bound weakly (4G2) or not at all (7D4), suggesting that they competed for similar epitopes (Fig 2f).

**Figure 4:**
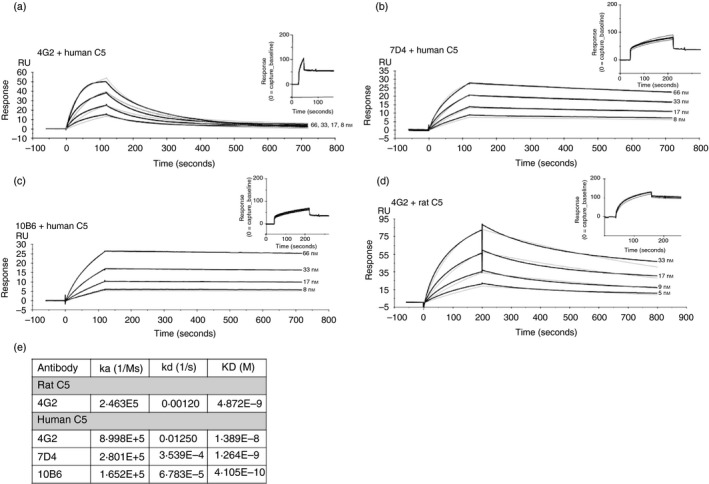
Binding kinetics of the novel monoclonal antibodies (mAb) to human and rat C5. Each mAb (4G2, 7D4, 10B6) was immobilized on the mouse IgG capture sensor chip (GE Healthcare) at approximately 60 RU (insets in each). C5 was flowed in HEPES‐buffered saline (HBS) at 66 to 8 nm for human and 33 to 4 nm for rat C5 and interactions with the immobilized mAb were analysed. Sensorgrams were collected and *K*_D_s were calculated using the Langmuir 1 : 1 binding model with RI values set to zero. Representative sensorgrams are shown with raw data in black lines and fitted data in grey (*n* = 3). (a) mAb 4G2 binding human C5; (b) mAb 7D4 binding human C5; (c) mAb 10B6 binding human C5; (d) mAb 4G2 binding rat C5; (e) summary table of *K*_D_s from the aggregate studies.

SPR analysis on immobilized antibody with human C5 flowed over (Fig. 4a–c) showed binding between human C5 and each of the novel mAb. The mAb 10B6 and 7D4, showed strong binding to human C5 in SPR analyses (*K*_D_ = 4·105 × 10^−10^, 1·264 × 10^−9^, respectively) with negligible off rates, suggesting that 10B6 and 7D4, respectively, targeting the *β* and *α* chains of C5, might be promising candidates for therapeutics. Binding of human C5 to 4G2 was relatively weak (*K*_D_ = 1·389 × 10^−8^; Fig. 4a); in contrast, binding of rat C5 to 4G2 was strong and stable (*K*_D_ = 4·872 × 10^−9^). The relatively slow off rate of rat C5 from mAb 4G2 suggested that this mAb offered promise for use *in vivo* in rats because 4G2 and C5 will form a stable complex, delivering prolonged inhibition. Technical issues precluded the analysis by SPR of 7D4 binding to rat C5.

### Induction of EAMG in rats and effect of mAb 4G2 on clinical disease and pathology

**Figure 5:**
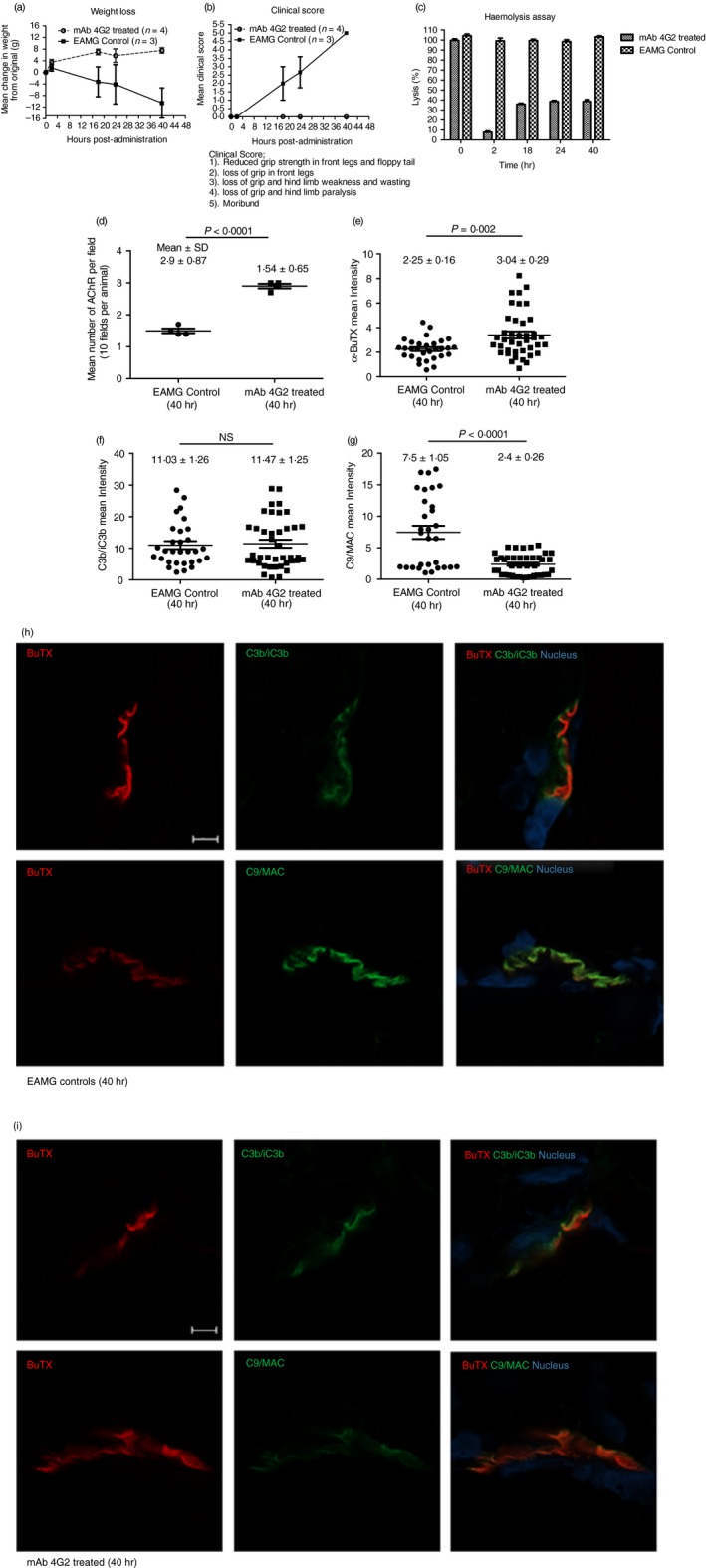
Therapeutic effect of monoclonal antibody (mAb) 4G2 in experimental autoimmune myasthenia gravis (EAMG). EAMG was induced in rats and weight loss (a) and clinical score (b) were monitored. Control animals (EAMG) rapidly developed weight loss and muscle weakness, reaching clinical scores of four or five and all were killed at 40 hr. The mAb 4G2‐treated animals were protected from disease and weight loss; all were killed at 40 hr. Serum lytic activity was measured at multiple time‐points (0, 2, 18, 24 and 40 hr) in each animal (c). Results are means of determinations from four 4G2‐treated and three isotype control‐treated EAMG animals and vertical bars represent SD. Soleus muscles were harvested at time of killing and snap frozen in OCT. Sections (10 μm) were stained for AChR with TRITC‐conjugated a‐BuTX and visualized by Zeiss Apotome microscope; AChR‐positive endplates were counted in 10 fields from each animal using imagej software (d). Mean fluorescence intensity of BuTx staining in the sections (mean pixel intensity values from imagej) was significantly higher in 4G2‐treated animals compared with controls (e; *P* = 0·002). Mean fluorescence intensity of C3b/iC3b was not significantly (NS) different between the two groups (f), while C9/MAC staining was markedly reduced in 4G2‐treated animals (g; *P* < 0·0001). Statistical significance was obtained by *t*‐test and *P* < 0·05 was considered as significant. Tissue sections from isotype control (h) or 4G2‐treated (i) animals were double‐stained for AChR together with anti‐C3b/iC3b (top panel) or C9/MAC (bottom panel) and imaged on a Zeiss confocal microscope. The scale bar is shown in the first plate; all images were captured at identical magnification.

Rats given mAb35 at 1 mg/kg intraperitoneally and an irrelevant isotype control mAb subcutaneously began to lose weight and show signs of hind limb weakness (Fig. 5a).^25^ Clinical symptoms, comprising limp tails, piloerection, hind limb weakness and reduced grip strength, were detectable in isotype control treated animals by 18 hr post‐induction, and all exhibited severe disease with hind limb weakness and/or partial paralysis, reaching clinical score 4 on a standardized scale (0, no disease; 1, reduced grip strength in front legs (can grip cage lid but cannot lift) and floppy tail; 2, loss of grip in front legs; 3, loss of grip and hind limb weakness and wasting; 4, loss of grip and hind limb paralysis; 5, moribund) by end point. In contrast, animals given mAb 4G2 subcutaneously at the time of disease induction continued to gain weight over the time course of the experiment and did not develop detectable weakness or other clinical manifestations for the duration of the experiment (Fig. 5b). Animals were killed using a Schedule 1 method when weight loss was equal to or exceeded 20% of original bodyweight, or when clinical score reached 4. CP haemolytic activity in serum was essentially absent at 2 hr post‐induction in 4G2‐treated animals but at later time‐points, residual haemolytic activity of approximately 35% of controls was detected (Fig. 5c), probably due to the high sensitivity of the haemolytic assay for residual C5.^20^ As expected, serum from the untreated control animals retained full haemolytic activity across the time course.

Soleus muscles were harvested at time of death (40 hr), sections (10 μm) were prepared, fixed in acetone and endplates were identified by staining AChR with *α*‐Bungarotoxin‐TRITC. Receptor numbers were quantified across 10 different representative fields in an automated imaging system and analysed using imagej software. The number of endplates in isotype control animals was significantly decreased compared with mAb 4G2‐treated animals (approximately twofold less; Fig. 5d); endplate numbers in the 4G2 group were not significantly different from numbers in naive animals (data not shown). Residual endplates were frequently fragmented in isotype control animals, whereas most endplates in 4G2‐treated animals displayed the typical continuous, linear character. The *α*‐Bungarotoxin fluorescence intensity was significantly higher in the 4G2‐treated animals (*P* = 0·002, Fig. 5e). Whereas C3 fragment staining intensity was similar between the groups, C9/MAC staining was reduced more than threefold in the 4G2‐treated group (*P* < 0·0001; Fig. 5f,g). Confocal analysis and co‐localization of C3b/iC3b and C9/MAC deposition at the endplate demonstrated strong and specific deposition of C3b/iC3b and MAC at the endplate in isotype control animals at 40 hr (Fig. 5h), whereas in 4G2‐treated animals, C3b/iC3b was deposited to a similar degree at endplates but C9/MAC deposition was weak or absent (Fig. 5i).

### mAb 4G2 but not mAb 7D4 and 10B6 block atypical cleavage of C5 by neutrophil elastase

**Figure 6:**
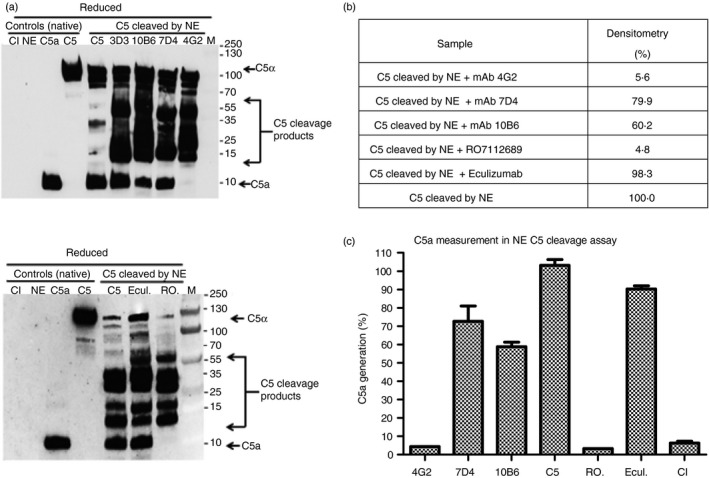
Impact of anti‐C5 monoclonal antibodies (mAb) on cleavage of C5 by neutrophil elastase. (a) Western blot of atypical cleavage of C5 by neutrophil elastase (NE). C5 was mixed with each mAb at 5× molar excess in HEPES‐buffered saline (HBS), NE was added at 420 nm, incubated and the reaction was stopped by addition of protease inhibitors. Samples (1 μg) were resolved on 4–20% SDS–PAGE gels under reducing (R) conditions and processed for WB to detect intact or cleaved C5 and C5a using an anti‐C5a mAb that also detects native C5*α* chain (Hycult; mAb 2942). C5*α* (115 000 MW), C5a (10·4 000 MW), CI; proteases cocktail inhibitors, Ecul.; Eculizumab, RO; RO7112689, 3D3; irrelevant non‐blocking anti‐C5 mAb. Note that numerous unidentified C5 cleavage products are present in all NE‐treated samples. (b) Densitometry analysis of the C5a band using imagej (expressed as % relative to the C5a generation in the absence of antibody; 100%) confirmed that mAb 4G2 and commercial mAb RO7112689 efficiently inhibited generation of C5a. (c) measurement of C5a generation by ELISA confirmed that mAb 4G2 and RO7112689 inhibited C5a generation while other mAb inhibited weakly. ELISA results are presented as percentage relative to C5a generation in the absence of any mAb (set as 100%; measured as 670 ng/ml in the ELISA). Results are representative of three independent experiments. The error bars are standard errors of triplicates.

To test whether any of the new mAb inhibited atypical cleavage of C5, C5 was incubated with NE with or without an excess of each of the mAb; C5a generation, detected by Western blot, was used as an index of C5 cleavage. Although C5 cleavage products were present in all cases, complete inhibition of NE‐mediated C5a generation was observed when mAb 4G2 was used, whereas 10B6 and 7D4 caused a partial inhibition of C5a generation compared with the no‐mAb control (Fig. 6a). When tested in this assay, RO7112689 completely inhibited C5a generation while Eculizumab had no effect on C5a production; an irrelevant non‐blocking anti‐C5 (3D3) also did not inhibit C5a generation (Fig. 6a). Densitometric analysis of the C5a band showed a ~95% inhibition of C5a generation by 4G2 and RO7112689, 40% by 10B6 and 20% by 7D4 compared with cleavage in the absence of antibody (Fig. 6b). These data were confirmed by ELISA; when compared with the amount of C5a generated by NE in the absence of antibody (670 ng/ml in the assay; 100%), the amount of C5a generated was reduced to ~4% by inclusion of 4G2 or RO7112689; 73% by 7D4; 59% by 10B6; Eculizumab did not inhibit C5 cleavage in this assay (Fig. 6c).

## Discussion

Twelve years ago, the first complement inhibitor, Eculizumab was approved for use in therapy of PNH. The discovery of Eculizumab and its dramatic success in this ultra‐rare disease catalysed Pharma interest in anti‐complement drugs, resulting in many new agents targeting different complement pathways being developed.^4^, ^14^, ^16^ Eculizumab is an effective terminal pathway blocker; however, for several years its use has been restricted to only two ultra‐rare diseases, PNH and aHUS, and latterly approved for MG treatment. In recent years, pathological roles of complement in more common conditions like MG, age‐related macular degeneration and neurodegeneration have been demonstrated, building a case for the broader use of anti‐complement drugs in the clinic.^27^, ^28^, ^29^ Cost and convenience are limiting factors in broadening anti‐complement drug use; current cost and dosing schedules for Eculizumab are incompatible with use in the treatment of common, chronic diseases. It is therefore imperative that new agents and approaches are brought to bear on C5 inhibition; increasing drug half‐life (and hence reducing dose) has been a first approach both with the Eculizumab‐derived ALXN1210 and the competitor mAb RO7112689, but other approaches, including small molecule inhibitors of C5,^30^ are in train. A second limiting factor is that the currently available agents are human‐specific, restricting proof‐of‐concept of efficacy of C5 inhibition in rodent disease models. The mAb BB5·1 has been widely used in mouse models,^31^, ^32^ and a single report described a blocking anti‐C5 mAb that was not further used,^33^ apart from these, no anti‐C5 mAb inhibiting in other animal species have been described.

Here we immunized C6‐deficient mice with C5b6 to develop functionally optimal mAb, selecting from the outset for function‐blocking activity. Of 864 000 hybridoma clones screened, 139 were ELISA‐positive, 12 of these were inhibitory, and three inhibitory clones were selected for full characterization. All mAb selected based on blocking activity bound native C5 and C5b6 (the immunogen), but not C6, in ELISA. Three of these human C5‐blocking mAb were selected for further characterization; testing for inhibition in other species showed that two of the mAb, 7D4 and 4G2 (both against C5*α* chain), inhibited C5 in rat, rabbit, guinea pig and mouse sera, whereas 10B6 (against C5*β* chain) was human‐specific. RO7112689 bound the C5*β* chain, as reported previously,^20^ and was a weak inhibitor of guinea pig and mouse C5 (Fig. 3), whereas Eculizumab bound C5*α* and is human‐specific.^34^ The critical residue in the Eculizumab binding site that dictates human specificity is Trp917, which is replaced by Ser in other species, including non‐human primates, rat, mouse, rabbit, guinea pig, sheep, pig and horse as reported,^34^, ^35^ and from our data base search (www.ncbi.nlm.nih.gov/protein). The fact that the two blocking anti‐C5*α* mAb 7D4 and 4G2 work across species suggests that they bind an epitope distinct from that in C5*α* bound by Eculizumab; this is supported by the demonstration that these mAb bind C5b6 (the immunogen) in the direct ELISA while Eculizumab does not. The mAb 4G2 detected C5 captured on Eculizumab in a sandwich assay, confirming that the epitopes are distinct; however, 7D4 did not bind in this assay, indicating that its epitope overlaps that of Eculizumab. When RO7112689 (C5*β*‐specific) was used as capture in the assay, both the C5*α*‐binding mAb detected the bound C5 as anticipated; in contrast, the C5*β*‐specific mAb 10B6 gave no signal, demonstrating that its epitope was masked by the capture mAb. Of note, RO7112689, like 10B6, also binds C5b6. These data indicate that the epitopes for these two mAb are overlapping; the fact that RO7112689 inhibits across species while 10B6 is human‐specific suggests that the epitopes are non‐identical. It is clear from the above findings, and from published work comparing tick‐derived C5 inhibitors with Eculizumab, that there are numerous sites on C5 where binding of an inhibitor can effectively block function.^36^ This supports the suggestion that these diverse inhibitors act as conformation locks, preventing a structural ‘priming’ event that is necessary for cleavage of C5 by the convertase.

The mAb were also tested for their capacity to block the atypical cleavage of C5 by NE, a process that has been implicated in the generation of C5a and MAC at inflammatory sites such as in the rheumatoid joint and cystic fibrosis lung;^37^, ^38^, ^39^ mAb that inhibited this cleavage might therefore have additional value as therapeutics. The mAb 4G2 strongly inhibited NE‐mediated C5 cleavage and C5a generation *in vitro*, whereas the other two novel mAb weakly inhibited atypical C5 cleavage (Fig. 6). Eculizumab did not inhibit C5 cleavage by NE but RO7112689 effectively blocked generation of C5a by NE (Fig. 6c).

SPR analysis (Biacore) showed strong and stable binding of 7D4 and 10B6 to human C5 with dissociation rates and calculated affinities comparable to RO7112689^17^ (Fig. 4). In contrast, 4G2 binding to human C5 was much weaker – but this mAb bound rat C5 much more strongly than human (*K*_D_ 4·872 × 10^−9^ for rat and 1·389 × 10^−8^ for human) and with a slow off‐rate; these binding data support the functional assays where 4G2 was a more potent inhibitor of rat C5 than human (Fig. 1). The 4G2 mAb was tested *in vivo* as a prophylactic therapy in a model of MG, previously demonstrated to be dependent on MAC assembly in man and rodents and suppressed by terminal pathway deficiencies or inhibition.^24^, ^26^ Although control animals developed severe muscle weakness and weight loss, mAb 4G2‐treated rats were protected from disease and weight loss (Fig. 5a,b). Endplates in controls were reduced in number, fragmented and richly decorated with C3b/iC3b and C9/MAC, whereas in 4G2‐treated animals endplates were preserved in number and integrity; although endplates were strongly C3b/iC3b‐positive in these animals, C9/MAC deposition was markedly reduced or absent (Fig. 6). Although mAb 4G2 proved to be an efficient blocker of EAMG, assays of serum haemolytic activity showed ~35% residual lysis after the 2 hr time‐point in 4G2‐treated animals. We have previously demonstrated that as little as 2 ng/ml C5 added back to undiluted C5‐depleted serum is sufficient to cause measurable lysis, and 50% lysis in the assay was attained at 2 μg/ml C5 added back.^20^ These findings illustrate the exquisite sensitivity of the haemolytic assay and show that it is not a good predictor of efficacy of C5 inhibition in disease. We have not yet tested this mAb for amelioration of established disease in the EAMG model, which would be difficult given the acute nature of the model, or in more chronic disease models; such studies are planned in the future.

The novel mAb described here are efficient blockers of human C5, with binding sites and other properties distinct from existing anti‐C5 mAb. All three mAb bind C5b6, used as the immunogen, and block reactive lysis, a property absent from Eculizumab that may provide a therapeutic advantage. Notably, two of the mAb inhibit C5 in common laboratory animal species, raising the prospect of their use as tools in proof‐of‐concept studies, illustrated here using EAMG as an example. One of the new mAb was a strong inhibitor of elastase‐mediated C5 cleavage, raising the possibility of testing the impact of blocking atypical C5 cleavage in inflammatory diseases where neutrophil activation predominates in rodents or man.

## Author contributions

WMZ performed all the laboratory analyses and wrote the first draft of the manuscript; PRT provided critical expertise on animal immunization and antibody clone screening; BPM conceived and planned the study and oversaw the data handling and manuscript preparation. All authors contributed to and have approved the final manuscript.

## Disclosure

BPM has provided advice on complement to Roche and is a consultant to GlaxoSmithKline; all fees are paid to Cardiff University. Other authors declared no potential conflicts of interest with respect to the research, authorship and/or publication of this article.

## References

[1] Phieler J , Garcia‐Martin R , Lambris JD , Chavakis T . The role of the complement system in metabolic organs and metabolic diseases. Semin Immunol 2013; 25:47–53. 23684628 10.1016/j.smim.2013.04.003 PMC3734549

[2] Stephan AH , Barres BA , Stevens B . The complement system: an unexpected role in synaptic pruning during development and disease. Annu Rev Neurosci 2012; 35:369–89. 22715882 10.1146/annurev-neuro-061010-113810

[3] Lubbers R , van Essen MF , van Kooten C , Trouw LA . Production of complement components by cells of the immune system. Clin Exp Immunol 2017; 188:183–94. 28249350 10.1111/cei.12952 PMC5383442

[4] Morgan BP , Harris CL . Complement, a target for therapy in inflammatory and degenerative diseases. Nat Rev Drug Discov 2015; 14:857–77. 26493766 10.1038/nrd4657 PMC7098197

[5] Edwards AO , Ritter R , Abel KJ , Manning A , Panhuysen C , Farrer LA . Complement factor H polymorphism and age‐related macular degeneration. Science 2005; 308:421–4. 15761121 10.1126/science.1110189

[6] Hageman GS , Anderson DH , Johnson LV , Hancox LS , Taiber AJ , Hardisty LI *et al* A common haplotype in the complement regulatory gene factor H (HF1/CFH) predisposes individuals to age‐related macular degeneration. Proc Natl Acad Sci USA 2005; 102:7227–32. 15870199 10.1073/pnas.0501536102 PMC1088171

[7] Haines JL , Hauser MA , Schmidt S , Scott WK , Olson LM , Gallins P *et al* Complement factor H variant increases the risk of age‐related macular degeneration. Science 2005; 308:419–21. 15761120 10.1126/science.1110359

[8] Klein RJ , Zeiss C , Chew EY , Tsai JY , Sackler RS , Haynes C *et al* Complement factor H polymorphism in age‐related macular degeneration. Science 2005; 308:385–9. 15761122 10.1126/science.1109557 PMC1512523

[9] Rother RP , Rollins SA , Mojcik CF , Brodsky RA , Bell L . Discovery and development of the complement inhibitor eculizumab for the treatment of paroxysmal nocturnal hemoglobinuria. Nat Biotechnol 2007; 25:1256–64. 17989688 10.1038/nbt1344

[10] Hillmen P , Young NS , Schubert J , Brodsky RA , Socié G , Muus P *et al* The complement inhibitor eculizumab in paroxysmal nocturnal hemoglobinuria. N Engl J Med 2006; 355:1233–43. 16990386 10.1056/NEJMoa061648

[11] Brodsky RA , Young NS , Antonioli E , Risitano AM , Schrezenmeier H , Schubert J *et al* Multicenter phase 3 study of the complement inhibitor eculizumab for the treatment of patients with paroxysmal nocturnal hemoglobinuria. Blood 2008; 111:1840–7. 18055865 10.1182/blood-2007-06-094136

[12] Wong EKS , Goodship THJ , Kavanagh D . Complement therapy in atypical haemolytic uraemic syndrome (aHUS). Mol Immunol 2013; 56:199–212. 23810412 10.1016/j.molimm.2013.05.224 PMC3899040

[13] Harris CL , Pouw RB , Kavanagh D , Sun R , Ricklin D . Developments in anti‐complement therapy; from disease to clinical trial. Mol Immunol 2018; 102:89–119. 30121124 10.1016/j.molimm.2018.06.008

[14] Harris CL . Expanding horizons in complement drug discovery: challenges and emerging strategies. Semin Immunopathol 2018; 40:125–40. 28986638 10.1007/s00281-017-0655-8 PMC5794834

[15] Morgan BP . Regulation of the complement membrane attack pathway. Crit Rev Immunol 1999; 19:173–98. 10422598

[16] Harris CL , Fraser DA , Morgan BP . Tailoring anti‐complement therapeutics. Biochem Soc Trans 2002; 30:1019–26. 12440965 10.1042/bst0301019

[17] Fukuzawa T , Sampei Z , Haraya K , Ruike Y , Shida‐Kawazoe M , Shimizu Y *et al* Long lasting neutralization of C5 by SKY59, a novel recycling antibody, is a potential therapy for complement‐mediated diseases. Sci Rep 2017; 7:1080. 28439081 10.1038/s41598-017-01087-7 PMC5430875

[18] Lee JW , Sicre de Fontbrune F , Wong Lee Lee L , Pessoa V , Gualandro S , Füreder W *et al* Ravulizumab (ALXN1210) vs eculizumab in adult patients with PNH naive to complement inhibitors: the 301 study. Blood 2018; 9:86136. 10.1182/blood-2018-09-876136 PMC6367644 30510080

[19] Sheridan D , Yu ZX , Zhang Y , Patel R , Sun F , Lasaro MA *et al* Design and preclinical characterization of ALXN1210: a novel anti‐C5 antibody with extended duration of action. PLoS ONE 2018; 13:e0195909. 29649283 10.1371/journal.pone.0195909 PMC5897016

[20] Zelek WM , Stott M , Walters D , Harris CL , Morgan BP . Characterizing a pH‐switch anti‐C5 antibody as a tool for human and mouse complement C5 purification and cross‐species inhibition of classical and reactive lysis. Immunology 2018; 155:396–403. 29981529 10.1111/imm.12982 PMC6187208

[21] Köhler G , Milstein C . Continuous cultures of fused cells secreting antibody of predefined specificity. J Immunol 1975; 174:2453–5. 15728446

[22] Orren A , Wallace ME , Hobart MJ , Lachmann PJ . C6 polymorphism and C6 deficiency in site strains of the mutation‐prone Peru‐Coppock mice. Comp Inflamm 1989; 6:295–6.

[23] Zelek WM , Harris CL , Morgan BP . Extracting the barbs from complement assays: identification and optimisation of a safe substitute for traditional buffers. Immunobiology 2018; 22:744–9. 10.1016/j.imbio.2018.07.016 30033110

[24] Ingram G , Hakobyan S , Hirst CL , Harris CL , Loveless S , Mitchell JP . Systemic complement profiling in multiple sclerosis as a biomarker of disease state. Mult Scler 2012; 18:1401–11. 22354735 10.1177/1352458512438238 PMC3697901

[25] Chamberlain‐Banoub J , Neal JW , Mizno M , Harris CL , Morgan BP . Complement membrane attack is required for endplate damage and clinical disease in passive experimental myasthenia gravis in Lewis rats. Clin Exp Immunol 2006; 146:278–86. 17034580 10.1111/j.1365-2249.2006.03198.x PMC1942064

[26] Morgan BP , Chamberlain‐Banoub J , Neal JW , Song W , Mizuno M , Harris CL . The membrane attack pathway of complement drives pathology in passively induced experimental autoimmune myasthenia gravis in mice. Clin Exp Immunol 2006; 146:294–302. 17034582 10.1111/j.1365-2249.2006.03205.x PMC1942050

[27] Dhillon S . Eculizumab: a review in generalized myasthenia gravis. Drugs 2018; 78:367–76. 29435915 10.1007/s40265-018-0875-9 PMC5845078

[28] Lu F , Liu S , Hao Q , Liu L , Zhang J , Chen X *et al* Association between complement factor C2/C3/CFB/CFH polymorphisms and age‐related macular degeneration: a meta‐analysis. Genet Test Mol Biomarkers 2018; 22:526–40. 30179527 10.1089/gtmb.2018.0110

[29] Morgan BP . Complement in the pathogenesis of Alzheimer's disease. Semin Immunopathol 2018; 40:113–24. 29134267 10.1007/s00281-017-0662-9 PMC5794825

[30] Ricardo A , Arata M , DeMarco S , Dhamnaskar K , Hammer R , Fridkis‐Hareli M *et al* Preclinical evaluation of RA101495, a potent cyclic peptide inhibitor of C5 for the treatment of paroxysmal nocturnal hemoglobinuria. Blood 2015; 126:939.26065653

[31] Copland DA , Hussain K , Baalasubramanian S , Hughes TR , Morgan BP , Xu H *et al* Systemic and local anti‐C5 therapy reduces the disease severity in experimental autoimmune uveoretinitis. Clin Exp Immunol 2010; 159:303–14. 20002447 10.1111/j.1365-2249.2009.04070.x PMC2819496

[32] Raedler H , Vieyra MB , Leisman S , Lakhani P , Kwan W , Yang M *et al* Anti‐complement component C5 mAb synergizes with CTLA4Ig to inhibit alloreactive T cells and prolong cardiac allograft survival in mice. Am J Transplant 2011; 11:1397–406. 21668627 10.1111/j.1600-6143.2011.03561.x PMC3128644

[33] Zhou Y , Gong B , Lin F , Rother RP , Medof ME , Kaminski HJ . Anti‐C5 antibody treatment ameliorates weakness in experimentally acquired myasthenia gravis. J Immunol 2007; 179:8562–7. 18056404 10.4049/jimmunol.179.12.8562

[34] Schatz‐Jakobsen JA , Zhang Y , Johnson K , Neill A , Sheridan D , Andersen GR . Structural basis for eculizumab‐mediated inhibition of the complement terminal pathway. J Immunol 2016; 197:337–44. 27194791 10.4049/jimmunol.1600280

[35] Brachet G , Bourquard T , Gallay N , Reiter E , Gouilleux‐Gruart V , Poupon A *et al* Eculizumab epitope on complement C5: progress towards a better understanding of the mechanism of action. Mol Immunol 2016; 77:126–31. 27497837 10.1016/j.molimm.2016.07.016

[36] Jore MM , Johnson S , Sheppard D , Barber NM , L YI , Nunn MA *et al* Structural basis for therapeutic inhibition of complement C5. Nat Struct Mol Biol 2016; 23:378–86. 27018802 10.1038/nsmb.3196 PMC5771465

[37] Giles JL , Choy E , van den Berg C , Morgan BP , Harris CL . Functional analysis of a complement polymorphism (rs17611) associated with rheumatoid arthritis. J Immunol 2015; 194:3029–34. 25725109 10.4049/jimmunol.1402956 PMC4367161

[38] Vogt W . Cleavage of the fifth component of complement and generation of a functionally active C5b6‐like complex by human leukocyte elastase. Immunobiology 2000; 201:470–7. 10776801 10.1016/S0171-2985(00)80099-6

[39] Fick RB , Robbins RA , Squier SU , Schoderbek WE , Russ WD . Complement activation in cystic fibrosis respiratory fluids: *in vivo* and *in vitro* generation of C5a and chemotactic activity. Pediatr Res 1986; 20:258–1268. 10.1203/00006450-198612000-00014 3540828

